# Supplementary material for: Crawling and Gliding: A Computational Model for Shape-Driven Cell Migration
Source: PLoS Comput Biol. 2015 Oct 21;11(10):e1004280. doi: 10.1371/journal.pcbi.1004280 (PMC4619082; doi:10.1371/journal.pcbi.1004280)
Supplement: S1 Code — (ZIP) [file pcbi.1004280.s012.zip › release/tst/doc/html/functions_func.html]

Tissue Simulation Toolkit: Class Members - Functions


Tissue Simulation Toolkit
 0.1.4.1


- Main Page
- Namespaces
- Classes
- Files

- Class List
- Class Hierarchy
- Class Members

- All
- Functions
- Variables
- Related Functions

- a
- b
- c
- d
- e
- f
- g
- i
- l
- m
- n
- o
- p
- q
- r
- s
- t
- w
- x
- y
- z
- ~

### - a -

- AbsorbingBoundaries()
  : PDE
- AddCell()
  : CellularPotts
- AddToGrad()
  : Cell
- addtoValue()
  : PDE
- AliveP()
  : Cell
- AllocateSigma()
  : CellularPotts
  , PDE
- AmoebaeMove()
  : CellularPotts
- Apoptose()
  : Cell
- Area()
  : Cell
  , Dish

### - b -

- BaseInitialisation()
  : CellularPotts
- BeginScene()
  : Graphics
  , QtGraphics
  , X11Graphics

### - c -

- Cell()
  : Cell
- CellBirth()
  : Cell
- CellDensity()
  : CellularPotts
- CellGrowthAndDivision()
  : Dish
- CellularPotts()
  : CellularPotts
- ChangeTitle()
  : X11Graphics
- CleanUp()
  : Parameter
- ClearGrad()
  : Cell
- ClearGrads()
  : Dish
- ClearImage()
  : QtGraphics
  , X11Graphics
- ClearJ()
  : Cell
- ClickCell()
  : Info
- Colour()
  : Cell
- ColourOfBirth()
  : Cell
- Compactness()
  : CellularPotts
- ConstructInitCells()
  : CellularPotts
- ConstructorBody()
  : Dish
- ContourPlot()
  : PDE
- CountCells()
  : Dish
- CropSize()
  : X11Graphics

### - d -

- DateOfBirth()
  : Cell
- Daughter()
  : Cell
- DecrementTargetArea()
  : Cell
- Diffuse()
  : PDE
- Dir()
  : Dir
- Dish()
  : Dish
- DivideCells()
  : CellularPotts
- DrawConvexHull()
  : CellularPotts

### - e -

- EndScene()
  : Graphics
  , QtGraphics
  , X11Graphics
- EnergyDifference()
  : Cell

### - f -

- Field()
  : Graphics
  , X11Graphics
- FindCellDirections()
  : CellularPotts
- Flush()
  : X11Graphics

### - g -

- getCell()
  : CellularPotts
  , Dish
- GetChemAmount()
  : PDE
- GetGrad()
  : Cell
- GetJ()
  : Cell
- getTau()
  : Cell
- GetXYCoo()
  : Graphics
  , QtGraphics
  , X11Graphics
- GradC()
  : PDE
- GradX()
  : Cell
- GradY()
  : Cell
- GrowAndDivideCells()
  : CellularPotts
- GrowInCells()
  : CellularPotts

### - i -

- IncrementTargetArea()
  : Cell
- Info()
  : Info
- Init()
  : Dish

### - l -

- Layers()
  : PDE
- Length()
  : Cell
- Line()
  : Graphics
  , QtGraphics
  , X11Graphics

### - m -

- MapColour()
  : PDE
- Mass()
  : CellularPotts
- Max()
  : PDE
- MaxSigma()
  : Cell
- MeanCellArea()
  : CellularPotts
- MeasureCellSize()
  : Cell
- MeasureChemConcentrations()
  : Dish
- Menu()
  : Info
- Min()
  : PDE
- Mother()
  : Cell

### - n -

- NoFluxBoundaries()
  : PDE

### - o -

- operator=()
  : Cell

### - p -

- Parameter()
  : Parameter
- PDE()
  : PDE
- PeriodicBoundaries()
  : PDE
- Plot()
  : CellularPotts
  , Dish
  , PDE
- PlotSigma()
  : CellularPotts
- PlotVectorField()
  : PDE
- Point()
  : Graphics
  , Point
  , QtGraphics
  , X11Graphics
- PrintInertia()
  : Cell

### - q -

- QtGraphics()
  : QtGraphics

### - r -

- Read()
  : Parameter
- ReadZygotePicture()
  : CellularPotts
- RecoverTitle()
  : X11Graphics
- RenormPolarVec()
  : Cell
- Replace()
  : CellularPotts
- ReplaceBeast()
  : X11Graphics
- ResetTargetLengths()
  : CellularPotts
- resizeEvent()
  : QtGraphics

### - s -

- SearchNandPlot()
  : CellularPotts
- SearchNeighbours()
  : CellularPotts
- Secrete()
  : PDE
- SetCellOwner()
  : Dish
- SetColour()
  : Cell
- SetGrad()
  : Cell
- SetJ()
  : Cell
- SetRandomTypes()
  : CellularPotts
- SetTargetArea()
  : Cell
- SetTargetLength()
  : Cell
- setTau()
  : Cell
- setValue()
  : PDE
- ShowDirections()
  : CellularPotts
- Sigma()
  : Cell
  , CellularPotts
  , PDE
- SimulationDone()
  : QtGraphics
- SizeX()
  : CellularPotts
  , Dish
  , PDE
- SizeY()
  : CellularPotts
  , Dish
  , PDE

### - t -

- TargetArea()
  : Cell
  , Dish
- TargetLength()
  : Cell
- TheTime()
  : PDE
- ThrowInCells()
  : CellularPotts
- Time()
  : CellularPotts
  , Dish
- TimesDivided()
  : Cell
- TimeStep()
  : Graphics
  , QtGraphics
  , X11Graphics
- TimeStepWrap()
  : QtGraphics

### - w -

- Write()
  : Graphics
  , Parameter
  , QtGraphics
  , X11Graphics
- WriteCOM()
  : Info

### - x -

- X11Graphics()
  : X11Graphics
- XField()
  : Graphics
  , QtGraphics
  , X11Graphics

### - y -

- YField()
  : Graphics
  , QtGraphics
  , X11Graphics

### - z -

- ZygoteArea()
  : CellularPotts
  , Dish

### - ~ -

- ~Cell()
  : Cell
- ~CellularPotts()
  : CellularPotts
- ~Dish()
  : Dish
- ~Graphics()
  : Graphics
- ~Parameter()
  : Parameter
- ~PDE()
  : PDE
- ~QtGraphics()
  : QtGraphics
- ~X11Graphics()
  : X11Graphics


---

Generated on Thu Aug 14 2014 22:04:02 for Tissue Simulation Toolkit by  

 1.8.6
